# Cell Division Site Placement and Asymmetric Growth in Mycobacteria

**DOI:** 10.1371/journal.pone.0044582

**Published:** 2012-09-10

**Authors:** Graham Joyce, Kerstin J. Williams, Matthew Robb, Elke Noens, Barbara Tizzano, Vahid Shahrezaei, Brian D. Robertson

**Affiliations:** 1 MRC Centre for Molecular Bacteriology and Infection, Department of Medicine, Imperial College, London, United Kingdom; 2 Department of Mathematics, Imperial College, London, United Kingdom; 3 European Molecular Biology Laboratory, Hamburg, Germany; Loyola University Medical Center, United States of America

## Abstract

Mycobacteria are members of the actinomycetes that grow by tip extension and lack apparent homologues of the known cell division regulators found in other rod-shaped bacteria. Previous work using static microscopy on dividing mycobacteria led to the hypothesis that these cells can grow and divide asymmetrically, and at a wide range of sizes, in contrast to the cell growth and division patterns observed in the model rod-shaped organisms. In this study, we test this hypothesis using live-cell time-lapse imaging of dividing *Mycobacterium smegmatis* labelled with fluorescent PBP1a, to probe peptidoglycan synthesis and label the cell septum. We demonstrate that the new septum is placed accurately at mid-cell, and that the asymmetric division observed is a result of differential growth from the cell tips, with a more than 2-fold difference in growth rate between fast and slow growing poles. We also show that the division site is not selected at a characteristic cell length, suggesting this is not an important cue during the mycobacterial cell cycle.

## Introduction

Cell growth and division are fundamental processes to all life and contribute to the morphological diversity observed across the prokaryote kingdom. Investigations into cell growth and division in bacteria have largely concentrated on a few model organisms, including *Bacillus subtilis* and *Escherichia coli* (see [1] for a recent review). Bacterial shape is determined and maintained by the rigidity of the peptidoglycan cell wall [2]–[4], the growth of which is controlled by directing peptidoglycan synthesis to specific sites within the cell [5]. These two rod-shaped bacteria control growth and division by similar mechanisms, using MreB to spatially regulate peptidoglycan synthesis [6]; while the Min proteins and nucleoid occlusion proteins ensure division occurs at mid-cell [7]–[9]. MreB is absent from mycobacteria and corynebacteria but widespread amongst other rod-shaped bacteria [10]. Previously thought to polymerize into a helical structure along the length of the cell to act as a scaffold for the peptidoglycan synthesis machinery [6], MreB has recently been shown to form mobile, fragmented elongation complexes that insert new peptidoglycan [11], [12].

The actinomycetes make up a morphologically diverse family including filamentous, coccoid, rod-shaped and fruiting-body producing bacteria, many of which display unique and complex life cycles [13], [14]. Mycobacteria and corynebacteria are both classified as rod-shaped but lack many of the cell division and growth systems identified as important for model rod-shaped organisms such as *E. coli* and *B. subtilis* [13], [14]. In contrast, the ability of mycobacteria to grow and divide asymmetrically at a wide range of sizes and ‘snap’ into a characteristic V-shape upon division has been postulated, suggesting a unique underlying mechanism controlling these important processes [15].

In contrast to *E. coli* and *B. subtilis,* rod-shaped actinomycetes, such as mycobacteria [16] and corynebacteria [17], [18], elongate apically by incorporating nascent peptidoglycan at the poles, rather than helically along the length of the cell. Mycobacteria and corynebacteria also do not appear to have many of the cell division systems found in other rod-shaped bacteria, which poses a number of questions about how mycobacteria regulate cell division and ensure the production of viable daughter cells [19], [20]. Cell elongation by polar growth poses problems in determining the mid-cell position, and could lead to asymmetric growth if elongation from opposing poles is not linked, some evidence of which is seen in electron microscopy studies of *Mycobacterium tuberculosis* [15].

Spatial regulation of peptidoglycan synthesis in mycobacteria and actinomycetes is controlled by DivIVA [21], [22], which localizes to regions of curved architecture, such as the cell poles [23]–[25]. DivIVA may act as an adapter protein for the cell wall biosynthesis machinery [26], including the Penicillin Binding Proteins (PBPs) that catalyse reactions involved in the final stages of peptidoglycan synthesis [27] and are the molecular targets for β-lactam antibiotics. PBP1a of *Corynebacterium glutamicum* has been shown to interact with DivIVA [28] and in both *C. glutamicum* and *B. subtilis* [29], [30] has been shown to localize to the cell poles and septa, consistent with it having roles in both the polymerization of lipid II by transglycosylation and linking glycans to peptides in other growing glycan strands [31]. In mycobacteria, PBP1 is proposed to interact with the resuscitation-promoting factor B interacting protein RipA, which also localizes at the poles and septa of diving cells. Binding of PBP1 to the RipA-RpfB complex inhibits its ability to hydrolyse peptidoglycan *in vitro*, suggesting there may be protein-protein interactions between enzymes with antagonistic functions that could regulate cell wall hydrolysis and synthesis [32].

Polymerization of the bacterial tubulin homologue FtsZ into a ring on the inner surface of the cell membrane represents the first stage in cell division, acting as a scaffold for the septation machinery. FtsZ polymerization, and therefore cell division in *E. coli* and *B. subtilis,* is regulated by two systems, nucleoid occlusion and the Min system. Nucleoid occlusion consists of nucleoid-associated FtsZ polymerization-inhibitors that prevent the cell division septum from forming around the bacterial chromosome [33], [34]. The bacterial chromosome in *E. coli* and *B. subtilis* is replicated at mid-cell and the daughter chromosomes are segregated towards the cell poles prior to division [35]. Therefore, the nucleoid is located at mid-cell during cell elongation, preventing the septum from forming in this region until the chromosome is segregated to the poles. Conversely, the Min system prevents the septum forming at the cell poles by localizing the FtsZ polymerization inhibitor, MinC to the poles [7], [36]–[38]. In *E. coli* and *B. subtilis* the combination of nucleoid occlusion and the Min system ensures that the Z-ring can only form at mid-cell when the cell has reached a characteristic length at which two nucleoids, segregated to the poles, leave a nucleoid free region at mid-cell, resulting in symmetrical division at a characteristic cell length [39]. Min homologues are absent in the corynebacterial and mycobacterial genomes, and nucleoid occlusion proteins have not yet been described, although two proteins potentially involved in septum formation have now been identified. The CrgA protein localized to the cell membrane, midcell and cell pole in *M. smegmatis* [40], and overexpression of Ssd protein in *M. smegmatis* and *M. tuberculosis* results in elongated cells, possibly by inhibiting septum formation [41]. If mycobacteria accurately select mid-cell for the division septa at a characteristic cell length an analogous system to the Min system may exist, but the question of how the cell division site is selected in these rod-shaped bacteria remains unanswered.

Previous work using static images [16] identified a pattern of mycobacterial cell sizes during growth that is inconsistent with a simple model in which cells double their length before division. Using fluorescent (FL) Vancomycin to label sites of peptidoglycan synthesis we observed an internal spot of FL-Vancomycin in addition to the polar spots, which was proposed to be associated with new cell wall septa [16]. However the internal spot does not always appear at the centre of the cell, which raises the question as to how, in the absence of a Min system and with actively growing cell poles, mycobacteria accurately identify mid-cell for septum positioning? Does inaccurate septum positioning lead to daughter cells of different size? Or are these previous observations possible artefacts of static imaging? To address these questions we used a combination of microscopy techniques, including time-lapse imaging, to study mycobacterial cell growth and division. Observations made using static phase-contrast microscopy were quantified using live-cell microscopy and fluorescently labelled PBP1a, which localizes to the poles and septa in actinomycetes [28], [31]. This demonstrates that despite the absence of an apparent Min system, the site for cell division can be selected accurately at mid-cell in a wide range of cell lengths. Unequal size daughter cells were observed and are a consequence of asymmetric growth from the cell poles, after the site for cell division has been selected.

## Results

### Cell Lengths and Internal Structures Stained with Fluorescent Vancomycin are more Variable in Mycobacteria than other Actinomycetes

**Figure 1:**
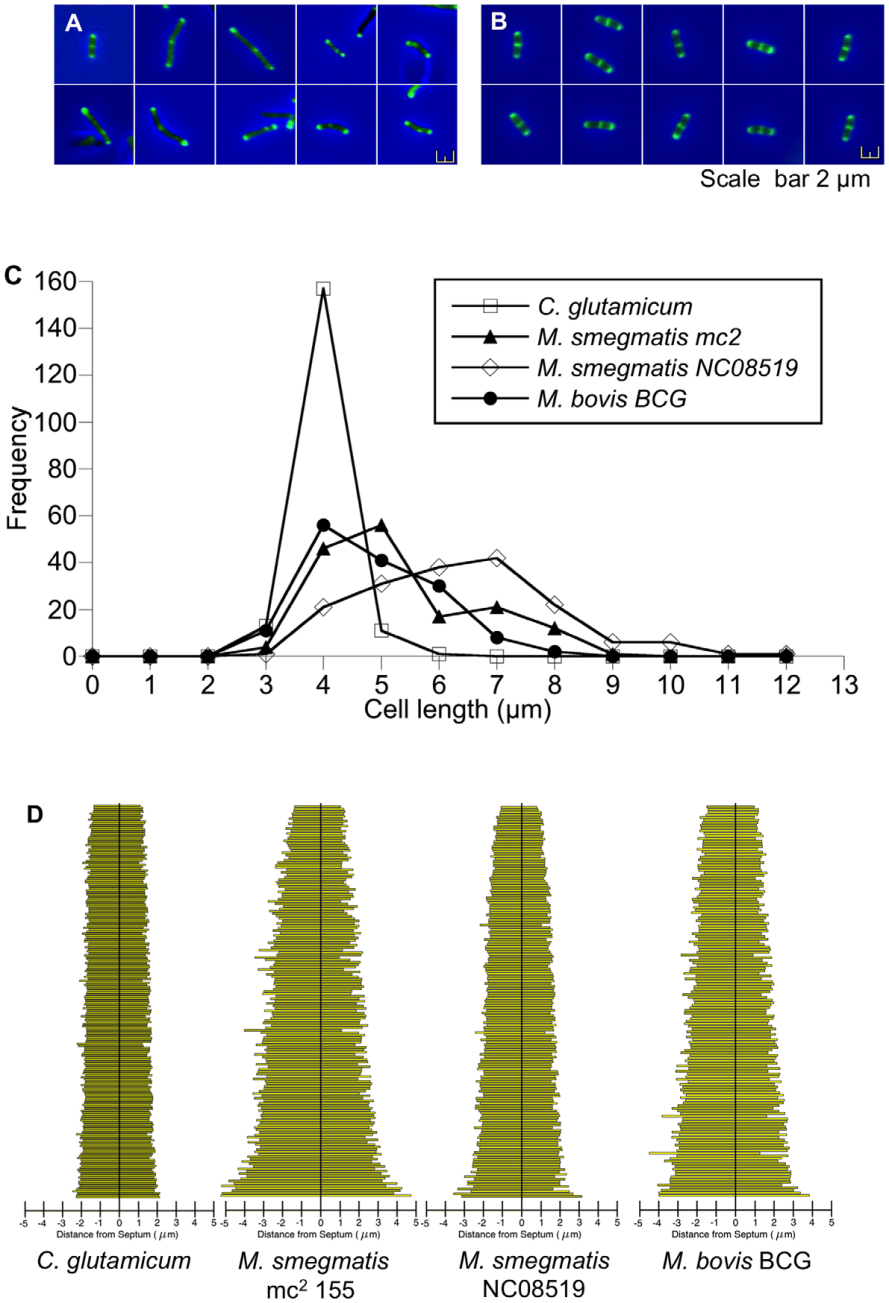
Internal spot placement and cell length are more variable in mycobacteria compared to *C. glutamicum* . (**A**) *M. smegmatis* mc^2^ 155 cells with two polar and one internal spots of VanBODIPY staining; note variable cell lengths and eccentric internal spot placement. (**B**) *C. glutamicum* cells with three spots are of similar size with a centrally located internal spot. Scale bar = 2 µm. (**C**) Plotting cell length versus frequency for 3-spot cells (n = 148) from all three species of bacteria, shows there is significantly more cell length variability in mycobacterial populations compared to *C. glutamicum* (p<0.01 Wilcoxon signed-rank test). (**D**) More than 95% of *C. glutamicum* cells contain an internal spot within the central 20% of the cell length, compared to only 70% of mycobacteria. Data for 3-spot cells was collected from three independent experiments.

To confirm our original observations of eccentrically placed internal vancomycin spots and variable cell lengths, we collected numerical data from *M. smegmatis* mc^2^155 (a transformable laboratory strain) [42], *M. smegmatis* NC08519 (a wild type strain from the NTCC), *M. bovis* BCG, and *C. glutamicum* stained with Van-BODIPY. We hypothesized that the internal spot marked the position of the new septum and that such cells should be about to divide, therefore we collected images from such cells and measured the position of the spots. Figure 1 shows that mycobacterial three-spot cells are more heterogeneous in appearance (Figure 1A), compared to *C. glutamicum* (Figure 1B), and measurement of these cells shows significantly greater variability in cell length in mycobacteria (Figure 1C): *M. smegmatis* mc^2^155 had a mean length of 4.8 µm, (standard deviation of 1.37 µm), and *M. bovis* BCG a mean length of 4.32 µm, (standard deviation of 1.1 µm), whereas *C. glutamicum* displayed a tight distribution of cell length with a standard deviation of 0.34 µm around the average cell length of 3.46 µm. Measurement of the position of the internal spot of Van-BODIPY staining shows that both mycobacteria and corynebacteria favour a mid-cell position, but there is more variation in mycobacteria with spots spread towards the poles of the cells: only 78% and 74% of spots are within the central 20 percentile for *M. smegmatis* and *M. bovis* BCG respectively, whereas 97% are within the central 20 percentile in *C. glutamicum* cells (Figure 1D). These results suggest that placement of the internal spot may be less accurate in mycobacteria compared to corynebacteria, and that mycobacteria divide at a wider range of cell lengths.

### Penicillin Binding Protein 1a Labels Cell Division Sites in Mycobacteria

**Figure 2:**
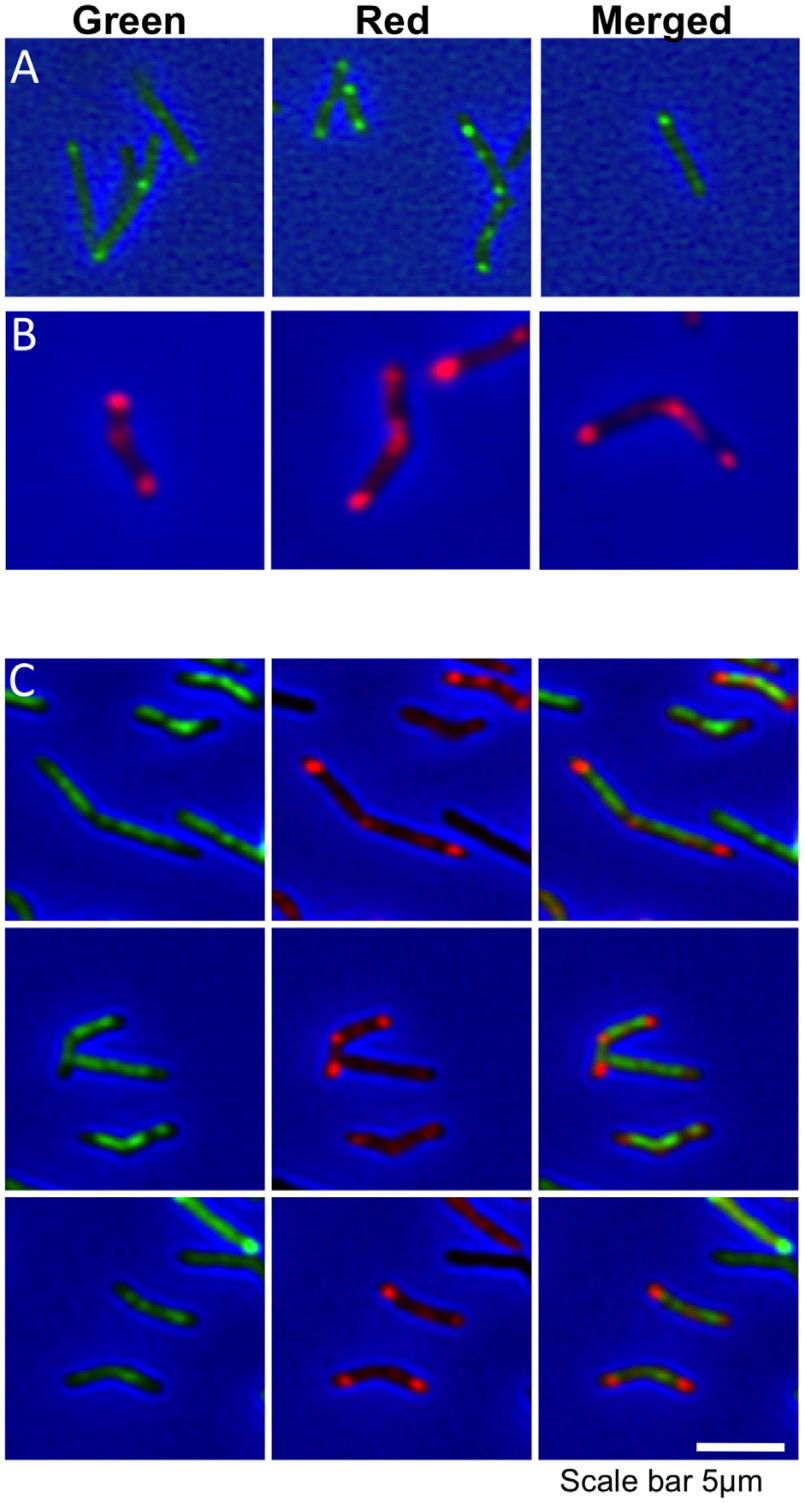
Co-localization of PBP1a and VanBODIPY in *M. smegmatis.* (**A**) Three fields of uninduced *M. smegmatis* mc^2^155 pMEND-PBP1a-mCherry stained with VanBODIPY display the characteristic polar and septal staining of nascent peptidoglycan (Green spots). (**B**) Induction of PBP1a-mCherry with 20 ng/ml Tc for 3.5 hr results in strong expression of red PBP1a-mCherry that localizes to the septum and poles, in a pattern similar to VanBODIPY staining (three fields of cells). (**C**) Expression of PBP1a-mCherry disrupts the localization of VanBODIPY staining, leaving diffuse green staining across the cell. The columns show the green, red and merged images respectively.

Vancomycin has a detrimental effect on bacterial growth at the concentrations required for effective staining (data not shown) and so it was not possible to follow live cells during division using this probe. FtsZ is the classical marker for septum formation, but has proved problematic in mycobacteria. Constitutive expression of FtsZ-GFP in a merodiploid is lethal, and expression from the inducible acetamidase promoter in an *ftsZ* mutant resulted in multiple FtsZ-GFP foci, filamentation and cell lysis [43], although recent reports have used tetracycline-inducible fluorescent FtsZ with some success [40], [44]. To check that we could reproduce the above observations we therefore developed a live-cell imaging method [45] that allowed us to follow the progress of septum markers during cell division. Penicillin binding protein 1a (PBP1a), which in *E. coli* polymerizes lipid II by transglycosylation and simultaneously attaches the growing glycan strand to monomeric peptides [31], was chosen as a suitable marker that displays polar and septal localization in corynebacteria [28] and *B. subtilis* [29], [30]. We first examined the co-localization of a Tetracycline-inducible *M. tuberculosis* PBP1a-mCherry fusion protein and Van-BODIPY in static *M. smegmatis*. In uninduced cells strong polar and septal staining with Van-BODIPY was observed, with barely visible levels of PBP1a-mCherry expression (Figure 2A). Induction of PBP1a-mCherry expression led to bright spots that localized to the poles and septa (Figure 2B). Addition of Van-BODIPY to these cells led to diffuse background staining across the cell surface, suggesting that both PBP1a and Van-BODIPY target the same sites of active peptidoglycan synthesis (Figure 2C).

**Figure 3:**
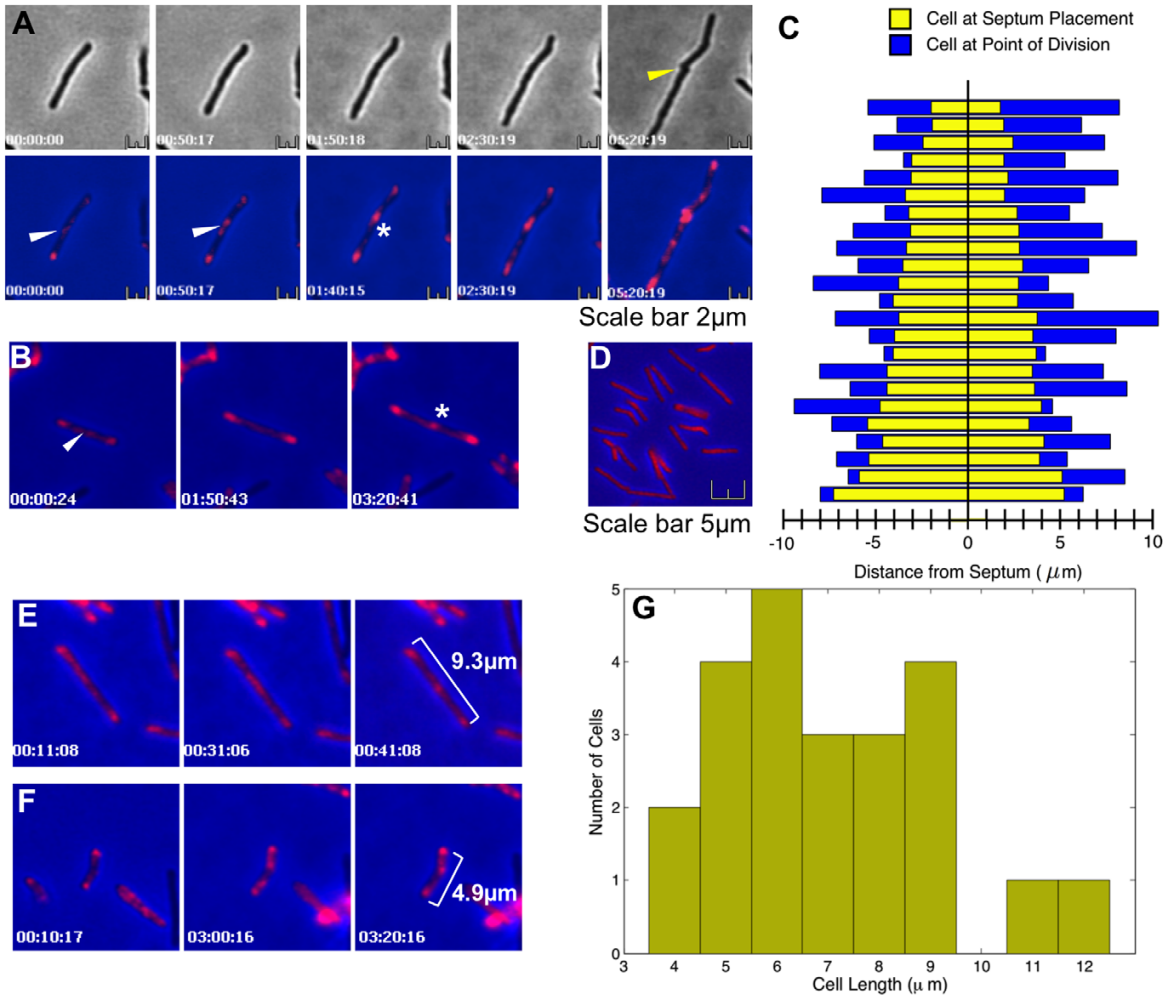
PBP1a-mCherry localizes centrally at septa that form future cell division sites, independently of cell length. PBP1a-mCherry expression in *M. smegmatis* mc^2^155 was induced with 20 ng/ml Tc for 3.5 hr cells and images were captured every 10 mins as described. Panels **A** and **B** are time series of images showing diffuse patches of staining at variable locations between the poles (white arrow). These eventually condensed into a central septal spot (white asterisk) around mid-cell. Cell envelope invagination and separation was not seen in the bright field images until 160 minutes after this condensation event (n = 10; yellow arrow). See File S1 and File S2 for complete movie sequences. The septal spots of PBP1a-mCherry form within the central 20% of the cell (Panel **C**; n = 23), without the outliers seen with Vancomycin staining. Panel D shows a control strain expressing mCherry alone. Septal spots are present in cells of various lengths (panels **E** and **F**), ranging between 4 and 12 µm (**G**), showing that cell division occurs at a wide range of cell lengths, indicating it is not a cue for placement of the new septum. See File S3 and File S4 for complete movie sequences.

Having established the pattern on PBP1a-mCherry localization in static cells, we then used live-cell imaging to follow the progress of PBP1a during cell division. Figures 3A and 3B show PBP1a-mCherry localized to future division sites (white asterisk), as detected in phase contrast images as a pinching of the cell envelope (yellow arrow). Initially a diffuse patch of PBP1a-mCherry was observed to temporarily condense at different points within the cell (white arrow), before finally condensing into a discrete septal spot, settling at the mid-point (Figure 3A and movie File S1). To determine whether *M. smegmatis* select the future division site precisely at mid-cell we measured the position of the PBP1a-mCherry septal spot relative to the poles (Figure 3B and movie File S2). Similar to the data in Figure 1, the septa are located towards mid-cell with 87% falling within the central 20 percentile (Figure 3C), but without the asymmetric outliers observed with Van-BODIPY staining (Figure 1). These outliers are probably the mobile PBP1a patches that precede condensation into a stable septal spot (See Figure 3A). Control strains expressing mCherry alone showed a diffuse pattern of staining across the cell with no localization (Figure 3D). These data also indicate that cell length is not a cue for division site placement in *M. smegmatis*, with a stable internal PBP1a-mCherry spot first appearing (Figures 3E and 3F, and movies File S3 and File S4) over a wide range of cell lengths from 3.75–12.5 µm (Figure 3G). This suggests that growth at the cell tips occurs at different rates and is not linked. Using the time-lapse image data collected we were able to determine the growth rates and found marked differences in the average exponential growth rate between the fast (1.66×10^−4^ min^−1^; SD = 2.39×10^−5^) and slow poles (7.87×10^−5^; min^−1^ SD = 8.80×10^−6^); the mean ratio of fast:slow rates is 2.386∶1. We observed a trend whereby 78% of cells grew faster on the side that was shorter after septum placement. There is also a weak correlation (Pearson −0.261, p = 0.2289) between the degree of asymmetry at septum placement and differential growth from the poles. However more data is needed to determine if these trends are statistically significant. Differences in spot intensity at the poles were observed, but no correlation was found with the growth rate (data not shown).

## Discussion

We have used a combination of live-cell imaging and fluorescent microscopy to show that the new septum in mycobacteria is placed accurately at mid-cell, and that the asymmetric division observed is a result of differential growth from the cell tips. Cell length does not appear to be an important cue for determining when cell division occurs. This is in contrast to previous work using static preparation of cells [16], but confirms a more recent report [46] using microfluidics-based live-cell imaging, in which the authors show that the heterogeneity in the population resulting from unipolar growth is linked to antibiotic sensitivity. We do not observe the unipolar growth reported, but see a >2-fold difference in the growth rate between fast and slow growing poles. The reasons for this disparity may lie in the methods and growth conditions used. Our current system is limited by the tendency of cells to grow in the z-plane, reducing the number of cells suitable for analysis.

We used a PBP1a-mCherry fusion as a marker in static cells to show it localizes to sites of new cell wall synthesis in the same way as vancomycin. In live cells vancomycin failed to localize when the PBP1a enzyme was overexpressed, presumably due to a decrease in the availability of the terminal D-ala-D-ala moieties it binds to. The PBP1a-mCherry merodiploid cells grew normally in the live-cell system compared to unlabelled cells [45] and showed no defects in broth culture when induced and non-induced cells were compared (data not shown). Taken together this indicates that the PBP1a fusion is enzymatically functional and likely to faithfully indicate the sites of active cell wall growth.

Using live-cell imaging we observed a diffuse cloud of labelled PBP1a moving within the growing cell. This eventually condensed at mid-cell at what would go on to be a division site, as evidenced by a pinching of the cell wall nearly 3 hours later. Measurement of the position of such true septal spots showed that, in contrast to the data obtained from vancomycin-labelled static cells, these spots localize accurately within the central 20% of the cell, as was observed for *C. glutamicum*. The movement of clouds of PBP1a within the cell prior to its final condensation provides a possible explanation for the outlying spots of vancomycin seen in static snapshots of cells, namely the movement of division complex components within the cell until the FtsZ ring completes and localizes at its final position. This may also be an artefact of the dead or dying mycobacterial cells used in static imaging, although this is not apparent in *C. glutamicum*. The use of PBP1a and live-cell imaging allowed us to identify the true septum and its final position within the cell, and demonstrate that mid-cell is selected with the same rigor as is seen in other organisms, but that is likely to be achieved by a different mechanism since it occurs at a range of cell lengths. We do not yet have the tools to address the timing of the various events leading to cell division, and do not know when PBP1a arrives at the septum in relation to FtsZ: if PBP1a arrives after FtsZ then we may be underestimating the precision of the placement of the septum at midcell.

In conclusion we have used live-cell imaging of mycobacteria to demonstrate that the site for cell division is selected accurately at mid-cell, and that this happens at a wide range of cell lengths. Unequal size daughter cells were observed and are a consequence of asymmetric growth from the cell poles after the site for cell division has been selected, and not as a result of off-centre positioning of the new septum. The question remains what benefit cells obtain from combining accurate midcell selection with asymmetric polar growth. There may be a selective benefit, as suggested by [46], in producing heterogeneously sized daughter cells which differ in their susceptibility to agents such as antibiotics. Programmed asymmetric division has been described for some alpha-proteobacteria (see [47] for a review), but it remains to be determined if that is the case here, and whether these mechanisms produce an advantageous distribution of cell sizes in the population.

## Materials and Methods

### Bacterial Strains and Growth conditions

*Mycobacterium smegmatis* mc^2^155 [42], *M. smegmatis* NC08519 (NTCC) and *Mycobacterium bovis* BCG Pasteur were grown aerobically at 37°C with shaking at 100 rpm (BCG) or 180 rpm (*M. smegmatis*) in Hartmans-de Bont minimal media [48]. *E. coli* MG1655 (ATCC No. 700926) was grown aerobically at 37°C with shaking at 200 rpm in Luria-Bertani (LB) broth (Merck). *C. glutamicum* (ATCC No. 13032) was grown aerobically at 30°C with shaking at 180 rpm in Tryptic Soy Broth (TSB; Sigma).

### Live-cell Time-lapse Video Microscopy

The bacterial strain for live-cell imaging was grown to mid-log phase, and imaging was carried out as described [45]. A 250 µl aliquot of the bacterial suspension was added to an uncoated glass-bottom-dish (Matek) and subsequently aspirated removing most of the liquid. The glass-bottom-dish was then filled with 3 ml standard growth broth containing 0.6% Noble agar (Sigma) at 37°C. This was supplemented with 20 ng/ml tetracycline for induction of PBP1a-mCherry. The agar was incubated at room temperature for 45 min to ensure complete solidification before mounting the specimen on the microscope within a Perspex housing at an ambient temperature of 37°C. The cells were viewed and images captured using a Zeiss Axiovert 200 inverted widefield microscope fitted with an EM-CCD (C9100-02) camera (Hammamatsu).

### Image Analysis

Basic cell image analysis was done using SimplePCI Compix software. Time-lapse analysis was carried out using Schnitzcell [49], [50] for segmentation. Pre-processing was performed in ImageJ to flatten the background noise by adjusting the colour levels. Fluorescent foci were detected through their maximal intensity pixel, recording their intensity, coordinates, and affiliation to its containing cell, enabling their tracking throughout the cellular lineages.

### Data Analysis

Growth rates were calculated using measurements of cell length at point of initial septum placement and at cell division and assuming an exponential growth rate. All mean and standard deviations were calculated using Excel, and the Pearson coefficient and corresponding p-value were calculated using Matlab. Plots were made using Kaleidagraph 4.1, Matlab, or the UsingR package.

### Vancomycin-BODIPY Staining

Fluorescent staining of nascent peptidoglycan synthesis was performed as described [16], [17]. Vancomycin-BODIPY (Molecular Probes) and unlabelled vancomycin each at 1 µg/ml (final vancomycin concentration was 2 µg/ml) were added directly to 1 ml mid-log phase culture and incubated for approximately half the cell generation time (90 min for *M. smegmatis*, 11 hr for *M. bovis* BCG and 45 min for *C. glutamicum*) under standard growth conditions. The cells were pelleted by centrifugation at 3000×g for 5 min, washed twice in 0.5 ml 0.05% Tween-80 in PBS, and finally resuspended in 50 µl 0.05% Tween-80 in PBS. Cell aliquots (10 µl) were spread on poly-L-lysine coated slides (BDH) using a sterile plastic loop and allowed to air dry.

Samples were mounted under a coverslip using Prolong Antifade reagent (Molecular probes) and examined using a 63× objective in a Zeiss Axiovert 200 inverted widefield microscope. Images were captured using an EM-CCD (C9100-02) camera (Hammamatsu) and cells measured using SimplePCI Compix software.

### Inducible Expression of PBP1a-mCherry

*M. tuberculosis* PBP1a (Rv0050) and mCherry were cloned into pMEND [51] to create a C terminal PBP1a-mCherry fusion. The entire coding region of Rv0050 lacking the stop codon was amplified by PCR using primers Rv0050_F (gcgcggatccgtggtgatcctgttgccgatgg) and Rv0050_R (gcgccatatgcggcggcggcgtgggagtc). This product was cloned as a *Bam*HI-*Nde*I fragment in front of the *mCherry* gene in plasmid pMEND-mCherry, and introduced into *M. smegmatis* mc^2^155 by electroporation. The resulting strain was grown to mid-log phase in Hartmans’s de Bont minimal media before the addition of 20 ng/ml tetracycline. For static imaging, the cell suspension was incubated for 3.5 hr, after which a 1 ml aliquot was pelleted at 3,000×g for 5 min and resuspended in 50 µl Hartmans’s de Bont minimal media. Cell aliquots (10 µl) were spread on poly-L-lysine coated slides (BDH) using a sterile plastic loop and allowed to air dry. Samples were mounted under a coverslip using Mowiol mounting media and examined immediately using a 63×objective in a Zeiss Axiovert 200 inverted widefield microscope. For live-cell time-lapse imaging, cells that had been induced for 3.5 hr with tetracycline were seeded into a glass-bottom-dish and imaged as described above.

## Supporting Information

File S1 **Time-lapse video sequence showing PBP1a-mCherry expression in** ***M. smegmatis*** **mc^2^155; images were captured every 10 mins.** Diffuse patches of staining appear at variable locations between the poles, and eventually condense into a central septal spot around mid-cell. Cell envelope invagination and separation was seen after this condensation event.
(MP4)

File S2 **Time-lapse video sequence showing PBP1a-mCherry expression in** ***M. smegmatis*** **mc^2^155; images were captured every 10 mins.** Diffuse patches of staining appear at variable locations between the poles, and eventually condense into a central septal spot around mid-cell. Cell envelope invagination and separation was seen after this condensation event.
(MP4)

File S3 **Time-lapse video sequence showing PBP1a-mCherry expression in** ***M. smegmatis*** **mc^2^155; images were captured every 10 mins.** Septal spots are present in cells of various lengths, indicating it is not a cue for placement of the new septum.
(MP4)

File S4 **Time-lapse video sequence showing PBP1a-mCherry expression in** ***M. smegmatis*** **mc^2^155; images were captured every 10 mins.** Septal spots are present in cells of various lengths, indicating it is not a cue for placement of the new septum.
(MP4)
